# Effectiveness of Different Training Modalities on Static Balance in Older Adults: A Systematic Review and Meta-Analysis

**DOI:** 10.3390/life13051193

**Published:** 2023-05-16

**Authors:** Noé Labata-Lezaun, Sergi Rodríguez-Rodríguez, Carlos López-de-Celis, Jacobo Rodríguez-Sanz, Max Canet-Vintró, Guillermo R.-Oviedo, Vanessa González-Rueda, Albert Pérez-Bellmunt

**Affiliations:** 1 Campus Sant Cugat, Universitat Internacional de Catalunya, 08195 Barcelona, Spain; nlabata@uic.es (N.L.-L.); carlesldc@uic.es (C.L.-d.-C.);; 2 Actium Functional Anatomy Research Group, Sant Cugat del Vallés, 08195 Barcelona, Spain; 3 Clínica SINAPSIS, Fisioterapia, Pensament i Moviment, 08720 Vilafranca del Penedès, Spain; 4 Fundació Institut Universitari per a la Recerca a l’Atenció Primària de Salut Jordi Gol i Gurina (IDIAPJGol), 08007 Barcelona, Spain

**Keywords:** balance, elderly people, older adults, training, exercise

## Abstract

(1) Background: aging is associated with functional changes such as balance, which plays a critical role in older adults. Physical exercise has been established as a factor capable of modulating these age-related alterations. (2) Methods: a meta-analysis of randomized clinical trials (RCTs) was conducted. The systematic search was performed in the PubMed/MEDLINE, Web of Science, the SPORTDiscus and Cochrane Library databases. Articles were included if participants were 65 years or older, healthy and performing resistance training, aerobic training, balance training or multicomponent training. Studies were excluded if there was a combination of training with other types of intervention. The protocol of this systematic review was published in the International Prospective Register of Systematic Reviews (PROSPERO) with the code CRD42021233252 (3) Results: the search strategy found a total of 1103 studies. After removing duplicates and the inclusion and exclusion criteria, eight articles were included in the meta-analysis, with a total of 335 healthy older adults analyzed. The results showed no significant differences between the intervention groups and the control groups after the exercise programs. (4) Conclusions: interventions based on different types of exercise improved static balance in elderly population, but without statistically significant difference in comparison with the control groups.

## 1. Introduction

The global population of older adults is growing rapidly [1,2] and at the same time, life expectancy is lengthening. Aging is associated with biological, structural, metabolic and neuroendocrine changes that influence the musculoskeletal system, leading to a decline in physical abilities, cognition and brain structure [2,3,4]. Recent studies agree that with aging, and induced by decreases in strength, endurance and consequently balance, there is also an increased rate of falls, which increases rapidly at 75 years of age [1,5,6,7,8,9].

In everyday life, balance plays a critical role for older adults in avoiding and adapting to the external environment that threatens postural stability [6]. In sports practice, the static balance could be fundamental [7]. Researchers highlight that postural sway during static standing increases with each decade of life in healthy adults aged 40 to 80 years [1]. They found that older adults rely on their visual field input to maintain balance. They further report that by the age of 70 years, the vestibular system shows a reduction, leading to less instability, and moreover there is a significant decrease in muscle strength leading to impaired balance [1,6,10].

In order to reduce these structural changes that accompany aging [3], physical exercise has been established as a factor capable of modulating age-related alterations, preventing muscle atrophy, maintaining cardiorespiratory capacity and cognitive function, improving metabolic activity and maintaining functionality [2,11]. Higher levels of physical exercise, such as multimodal training regimens that include strength, balance or general endurance tasks, may reduce overall morbidity and improve quality of life [5,12,13,14,15].

It has been described in the scientific literature that there was no consensus regarding which of the critical elements of motor control needed to be trained to improve balance [8]. Currently, numerous multicomponent interventions (MCT) have been proposed to improve balance in older adults, such as strength training, aerobic exercise, or specific flexibility training [16]. In addition, the latest international consensus on exercise in older adults [17] described MCT programs as beneficial strategies to improve balance among other domains [12,17]. Other recent guidelines on fall prevention in older adults recommend in their guidelines exercise training modes that are highly challenging for balance, such as resistance training in addition to balance training [18].

In line with this, in recent years and research, several rehabilitation modalities with digital platforms, such as exergames or Wii fit [19,20,21], based on physical-cognitive interaction through body movements, have emerged as promising approaches in the treatment of balance in older adults.

Although there is a large amount of scientific literature on therapeutic exercise and the benefits it brings, among other capacities, to balance in the elderly, there are many exercise modalities that have been proposed in recent years. The aim of this review is to evaluate, through an analysis of the scientific literature, which exercise modalities have the greatest benefits for balance in the elderly.

## 2. Materials and Methods

The present systematic review and meta-analysis was reported in accordance with the Preferred Reporting Items for Systematic Reviews and Meta-Analyses (PRISMA) statement [22]. The protocol of this systematic review was previously published in the International Prospective Register of Systematic Reviews (PROSPERO) with the code CRD42021233252.

### 2.1. Systematic Literature Search

First, a systematic literature search was conducted in the electronic search engines PubMed/MEDLINE and Web of Science, and in the SPORTDiscus and Cochrane Library databases until January 2023. Moreover, additional explorations were conducted weekly with automatic updates retrieved from the aforementioned databases. In addition, a manual search was conducted, and the bibliography of other similar systematic reviews was checked in order to find potential studies. The complete search strategies are available in the Supplementary Material S1.

In order to develop an accurate search strategy, the PICOS strategy was used [23]. Four domains were included. Population: healthy older adults; intervention: resistance training (RT), aerobic training (AT), balance training (BT) or multicomponent training (MCT), including at least 3 training modalities; comparison: no training; outcome: static balance; study design: randomized control trials (RCTs). Filters proposed by the Cochrane Collaboration for RCTs were also combined with the search strategy [24].

### 2.2. Selection Criteria

Inclusion criteria: randomized controlled trial study design, older adults aged >65, considered as healthy participants, intervention group with a RT, AT, BT or MCT, comparison group with no training, static balance as outcome and English language. Exclusion criteria: combination of training with other types of intervention.

### 2.3. Screening, Selection and Data Extraction Process

Once the systematic search was conducted, all titles, abstracts and full text were independently screened by two researchers (JRS and MCV) for potential inclusion. A third researcher (NLL) was consulted for a decision in case of discrepancy. Rayyan software (https://rayyan.qcri.org, accessed on 15 January 2023) was used in order to perform the screening process [25]. The following data were extracted from every included study: author’s last name, year of publication, sample size, sample characteristics (number of participants in each group, age and gender distribution), characteristics of the training (duration of the program, frequency, volume, intensity and exercise selection), control group characteristics, outcomes and main results. The data were extracted independently by two reviewers (NLL and CLdC).

### 2.4. Methodological Quality and Risk of Bias

Methodological quality and risk of bias of the included studies were independently evaluated by two authors (APB and NLL) using the Physiotherapy Evidence Database (PEDro) scale [26], and the Cochrane Risk of Bias (RoB2) assessment tool [27].

The PEDro scale was especially designed to assess the methodological quality of the physiotherapy studies. It was developed to assess 3 domains (external validity, internal validity and statistical reporting), and included the following 11 items: (1) eligibility criteria and source (2) random allocation, (3) concealed allocation, (4) baseline comparability, (5) blinding of participants, (6) blinding of therapists, (7) blinding of assessors, (8) adequate follow-up (>85%), (9) intention-to-treat analysis, (10) between-group statistical comparisons, (11) point measures and variability data. All items achieved are scored 1, and the total PEDro score is calculated by adding items 2 to 11, with a maximum score of 10. PEDro score <4 is considered “poor”; from 4 to 5 “fair”; from 6 to 8 “good” and 9 to 10 “excellent” [26].

RoB2 is a widely accepted tool in the biomedical scientific field, proposed by Cochrane Collaboration [27], to evaluate the potential risk of bias of a RCT. It is divided into six domains, involving the following 8 items: randomization sequence generation, allocation concealment, blinding participants, blinding therapist, blinding outcome assessor, incomplete outcome data, source of funding bias/selecting outcome reporting and other bias. Each item is categorized in 3 scores: low (green), unclear (yellow) or high (red) risk of bias.

### 2.5. Data Synthesis and Analysis

The Review Manager 5 software (Cochrane Collaboration, Oxford, UK) was used to perform the statistical analysis. As all outcomes were continuous, sample size, post-intervention means and standard deviation (SD) were extracted. Corresponding authors were contacted by e-mail if any data not reported in the paper were required.

The mean difference (MD) was chosen as the effect size, when studies used the same tool of measure. The standard mean difference (SMD) was chosen as the effect size when studies used different tools of measure. The effect size was expressed with a 95% confidence interval (95%CI). The inverse of the variance (IV) statistical test was used for the quantitative analysis, using a random-effects model to determine the overall effect size, as the number of included studies was considered small [28]. eStatistical significance was set at *p* < 0.05.

The heterogeneity was evaluated by using I^2^ statistics, and it was classified as low (I^2^ ≤ 25%), moderate (25 < I^2^ < 50%) or high (I^2^ ≥ 50%) [28]. When high heterogeneity (I^2^ > 50%) was found, a heterogeneity analysis using subgroups was performed. Finally, visual inspection of the funnel plots was performed to assess potential publication biases if the number of the included studies was more than 10 [29].

## 3. Results

### 3.1. Study Selection

**Figure 1:**
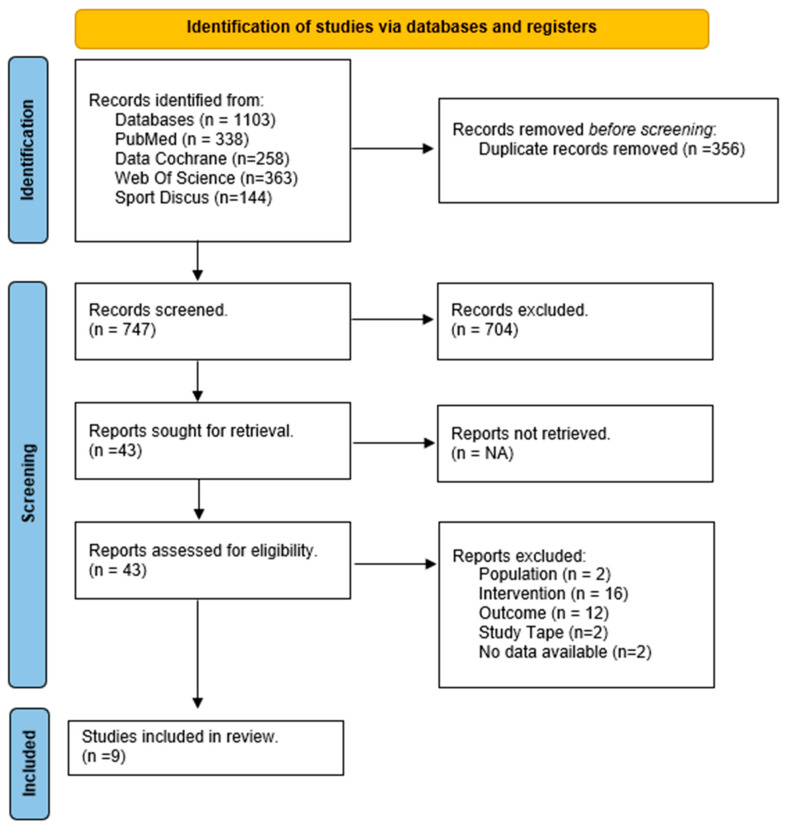
PRISMA 2020 flow diagram.

The search strategy found 1103 studies (Cochrane Library: 258; PubMed: 338; Web of Science: 363; SPORTDiscus: 144). After removing duplicates, a total of 747 studies were initially considered to be included. Seven hundred and four studies were excluded after screening the titles and abstracts for not meeting the inclusion criteria. After assessing the full text, nine studies met the inclusion criteria for the qualitative analysis, and 34 articles were excluded. One study dropped out from the meta-analysis for not presenting the required data, after contacting the corresponding author. Finally, eight articles were included in the quantitative synthesis, three for the RT intervention [30,31,32], three for the BT intervention [31,33,34] and two for the MCT intervention [4,35]. Figure 1 presents the PRISMA flow diagram [22].

### 3.2. Study Characteristics

Table 1 summarizes the sample characteristics of the nine included articles. A total of 335 elderly individuals were included in this systematic review. Although most of the articles analyzed both men and women, the distribution was greater for women. The sample size varied between 12 [36] and 55 [31] individuals, with an average age between 67.5 [30] and 81.5 years old [36]. In terms of the duration of the interventions, the training programs varied from 3 [36] to 48 weeks [35], with a frequency of two to three sessions per week. As for the control group, seven studies included a non-training group, one included educational session [31] and another one included a walking activity [32]. Only the studies by Cadore et al. [37] and Forte et al. [38] included a resistance-training comparison group. Finally, regarding the study variables, static balance was assessed with the one-legged stance test in five studies, with the Berg balance scale in two studies [33,36], with the Romberg test in one study [34], and with the short physical performance battery (balance test) in another study [4].

### 3.3. Methodological Quality

The average score for the PEDro scale was 5/10, which is considered as a “fair” methodological quality across the studies included in the systematic review. The main recurrent methodological problems were blinding of participants and therapists (0/9 studies); and blinding of assessors, concealed allocation and intention-to-treat analysis (2/9 studies) (Table 2) [26].

### 3.4. Risk of Bias

**Figure 2:**
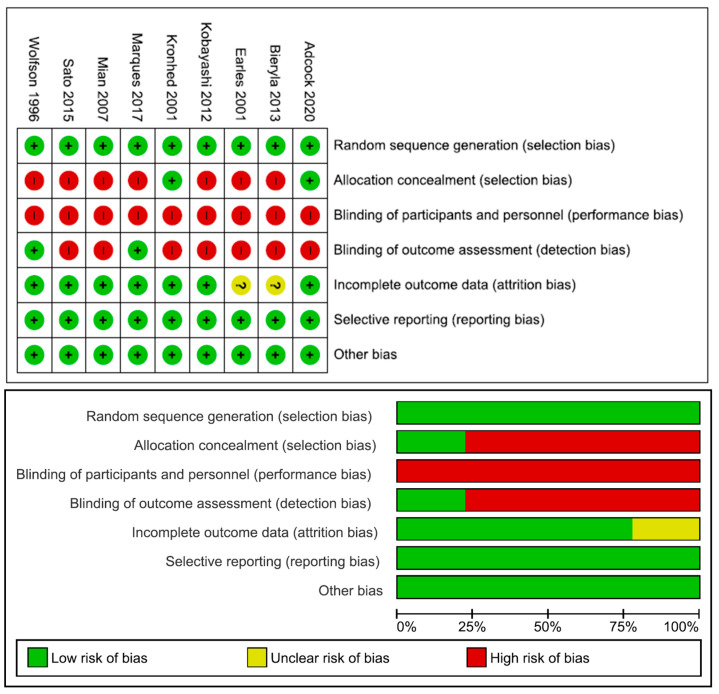
Risk of bias graph and summary [4,30,31,32,33,34,35,36,39].

Figure 2 shows the RoB 2 tool summary and graph. Again, a high risk of bias was found in terms of performance bias (9/9), selection bias (7/9) and detection bias (7/9). (+) signs in green indicates low risk of bias, (?) signs in yellow indicates unclear risk of bias, and red marks indicates high risk of bias.

### 3.5. Effectiveness of Interventions

#### 3.5.1. Resistance Training

**Figure 3:**
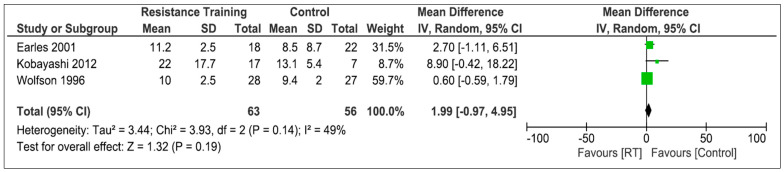
Effectiveness of resistance training on static balance [30,31,32].

Four articles reported results about resistance training [30,31,32,39]. The study conducted by Earles et al. [32] focused on speed-based strength training. Among their results, they found improvements in strength, but not in functional capacity, which included the static balance variable. In the studies carried out by Kobayashi et al. and Marques et al. [30,39], they found improvements in static balance after performing a specific strength training protocol with respect to the control group. Finally, in the study conducted by Wolfson et al. [31], non-significant improvements in static equilibrium were found. For methodological reasons when reflecting the results, and after contacting the corresponding author, the data obtained in the study of Marques et al. could not be included in the quantitative analysis, so three studies were included, with a total of 119 participants. Figure 3 shows the comparison between RT and the control group. Analysis shows an overall SMD of 1.99 [95%CI −0.97; 4.95] and an overall effect of Z = 1.32 (*p* = 0.19). The heterogeneity was considered moderate (I^2^ = 49%).

#### 3.5.2. Aerobic Training

In reference to aerobic training, only one article was included in the qualitative analysis. The study carried out by Marques et al. [39] found improvements in static balance with respect to the control group after an aerobic training program with an intensity from 50% to 85% of the reserve heart rate.

#### 3.5.3. Balance Training

**Figure 4:**
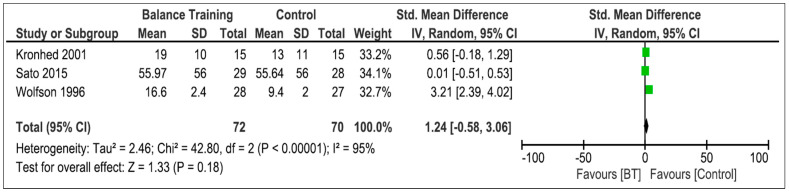
Effectiveness of balance training on static balance [31,33,34].

Four studies [31,33,34,36] evaluated the effectiveness of balance training in improving static balance. Only studies conducted by Bieryla et al. and Wolfson et al. reported significant improvements. For methodological issues when reporting the results, and after contacting the corresponding author, the data obtained in the study of Bieryla et al. could not be included in the quantitative analysis, so three studies were included, with a total of 119 participants. Figure 4 shows the comparison between BT and the control group. Analysis shows an overall SMD of 1.24 [95%CI −0.58; 3.06] and an overall effect of Z = 1.33 (*p* = 0.18). The heterogeneity was high (I^2^ = 95%).

#### 3.5.4. Multicomponent Training

**Figure 5:**
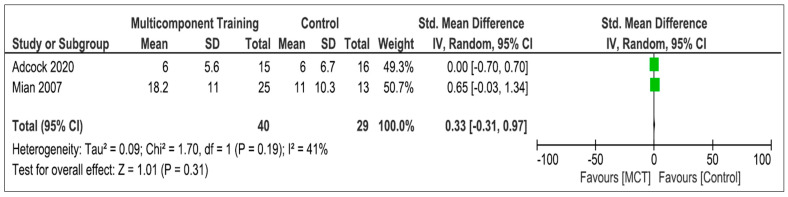
Effectiveness of multicomponent training on static balance [4,35].

Two studies [4,35] evaluated the effectiveness of MCT in improving static balance, without obtaining differences with respect to the control group in any of them. Sixty-nine participants were included in the meta-analysis. Figure 5 shows the comparison between MCT and the control group. Analysis shows an overall SMD of 0.33 [95%CI −0.31; 0.97] and an overall effect of Z = 1.01 (*p* = 0.31). The heterogeneity was moderate (I^2^ = 41%).

After analyzing the results of the different interventions, it has been observed that all the articles present moderate or high heterogeneity, and it can be concluded that none of the interventions present statistically significant differences with respect to their control groups.

## 4. Discussion

The present study aims to analyze the effects of different types of training such as aerobic, resistance, multicomponent and balance training on static balance in the healthy elderly population. This systematic review summarizes the findings from a total of eight studies and includes a total of 335 participants. Having described the results in the previous section, our review shows no significant statistical differences between any of the training modalities mentioned and their respective CGs, in terms of improvements in static balance.

### 4.1. Resistance Training

It is important to note that the heterogeneity between studies was moderate for resistance training (I^2^ = 49%). This could be explained by the difference in the years of publication of the studies analyzed by Earles et al., Kobayashi et al. and Wolfson et al., the difference in sampling and that the study by (Wolfson et al.) [31] adds balance training to their intervention.

The results of the present study are in agreement with those of previous studies (Knerl C et al.) [40] that found no significant improvement in balance after a resistance exercise programme compared to CG. Furthermore, Motalebi SA et al. [41] found significant improvements in lower limb strength and dynamic balance when applying a resistance programme, but static balance values measured with tandem stand (TS) and one leg stand (OLS) showed no improvement after 12 weeks. Schlicht et al. [42] also found no significant improvement in static balance after 8 weeks of intensive lower limb resistance training. This could be explained by the fact that several studies (Kobayashi et al., Schlicht et al. and Knerl C et al.) [30,40,43] present an intervention time shorter than 12 weeks, which is the minimum recommended time by Fragala MS et al. [44]. Still, more research should be carried out to analyze the effects of resistance training with durations shorter than 12 weeks.

### 4.2. Multi-Component Training

Multicomponent training has considerable adherence among older populations, being a modality that has the characteristic of encompassing different abilities [42]. Previous trials have highlighted the potential benefits of a multicomponent exercise programme (resistance, aerobic endurance, flexibility and balance exercises) of long duration (i.e., 6 months or more) on functional capacity in older populations [16].

After analyzing the results, we highlight that the heterogeneity between studies (Adcock et al. and Mian et al.) [4,35] was moderate for multicomponent training (I^2^ = 41%). This could be due to the difference in the year of publication between the studies, the type of intervention and the duration of the sessions.

In our review, no significant statistical differences in terms of improvement of physical abilities such as static balance were found between the intervention group and the CG after an MCT programme. In controversy with our results, another study by Sadjapong U et al. [45], where a multicomponent exercise programme was performed in older adults, found significant improvements in balance (BERG test and TUG test) compared to baseline (*p* < 0.001), compared to the CG. The study by Casas HA et al. [16] found significant differences after a multicomponent programme in older adults in Spain, with 0.86 points in the SPPB score (95%CI 0.32, 1.41; *p* < 0.01) after 1 month and 1.40 points (95%CI 0.82, 1.98; *p* < 0.001) after 3 months.

Previous trials [46] have highlighted the potential benefits of an MCT programme (resistance, endurance, flexibility and balance exercises) on functional capacity in older populations following exercise interventions of longer duration, i.e., 6 months or more. This could explain why there is no significant change in the results of the study by Adcock et al., as in their study, the intervention was performed without professional supervision from home and for a duration of less than 6 weeks. In the study by Mian et al., part of the intervention was also conducted from home, unlike the study by Sadjapong U et al. [45] where the 12-week multicomponent exercise intervention was guided and supervised by therapists. Recent studies [45] concur on the problems of older adults in finding strategies to increase their adherence and motivation to home-based exercise programs.

### 4.3. Balance Training

Balance is defined as the ability to maintain an upright posture during static and dynamic tasks and requires complex interactions between central factors such as vision, somatosensation, vestibular sensation, motor skills and musculature [40]. Intervention programs that incorporate balance-challenging exercises have been shown to reduce the risk of falls among older adults [21].

With regard to the characteristics of the different interventions, once the results have been analyzed, we have seen that there is heterogeneity of over 40% in all the variables studied, with the studies on balance training being those with the highest heterogeneity (I^2^ = 93%). This could be due to the fact that the three studies were published in very different years, the difference in sample size and that the study by Wolfson et al. also performed resistance training in addition to balance training.

In the study by Lopez JC et al. [47], they observed that both groups improved unipodal and bipodal balance with both eyes open and closed after the intervention on unstable platforms; both differences were statistically significant (*p* < 0.01). This study is in agreement with the balance improvements reported in our review study (Wolfson L et al.) [31], who performed balance and strength interventions on stable surfaces, including Ti-Chi methods. Javadpour S et al. and Sinaei E et al. [48,49] showed significant improvements in static balance and dynamic balance in the single-task (ST) and dual-task (DT) training groups after 6 weeks in the Javadpour S study and 4 weeks of training in the Sinaei E study, while no statistically significant changes were shown in the control group.

In our review, no statistically significant changes were observed between the balance exercise-based intervention and CG groups. Only Wolfson et al. showed significant improvements. This could be due to the fact that in the Wolfson et al. study, in addition to balance training, resistance training was also performed. Two studies (Sato K et al. and Kronhed ACG et al.) [33,34] did not show significant results compared to the control group in comparison to the other studies mentioned [47,48,49]. This could be explained by the fact that the studies by Sato K et al. and Kronhed ACG et al. did not use dual task exercises in their interventions and by the fact that the intervention by Sato K et al. used exergames in older adults, as it has been described [19] that research on persuasion in technology has not yet focused on older adults, which may not motivate or hinder their rehabilitation if carried out through exergames.

### 4.4. Aerobic Training

One of the reported benefits of aerobic activity is the improvement of balance and the reduction of the risk of falls and subsequent injuries caused by such an accident [50].

The study by Marques et al. found that aerobic exercise has a similar effect to that produced by strength training on static balance in older women aged 61–83 years, with respect to CG after an aerobic training programme, at an intensity of 50% to 85% of heart rate reserve. Spagnuolo et al. [51] and colleagues also found contrasting results with a strong correlation between the performance achieved on the incremental out-and-back walking test (ISWT) and the Berg balance scale in older adults, but aged 40–84 years (r = 0.61). The above results are consistent with the following study [52]: a programme that included dancing as an aerobic exercise for 1 h, three times per week, for 12 weeks, observed significant changes in static balance in women over 70 years of age. In addition, Vidarte-Claros JA et al. [53] showed changes in balance in older people who performed the exercise programme for 12 weeks, with similar effects on reducing the risk of falls.

In controversy, Hayashi D et al. [54], found no correlation between postural balance and exercise capacity in the whole group of older adults. This could be due to the fact that part of their sample were physically independent older adults according to the classification proposed by the Functional Status Spirduso.

### 4.5. Limitations of the Study

Regarding the limitations of this study, it is important to comment on the heterogeneity of the results of each study, as well as the intrinsic characteristics of the studies. It can be stated that the lack of homogeneity of the different studies, due to among other factors differences in the year of publication, sample size and type of intervention, may have contributed to show statistically non-significant results.

Furthermore, we are aware that the number of studies added to the review is small and that in one of the studies, the author did not provide us with the data. Regarding the characteristics of the sample, we note that although all the studies analyzed older adults, in some of the studies, the physical abilities of the participants varied, which may have increased the heterogeneity.

## 5. Conclusions

In view of our results, we can conclude that different modalities of training improve functional capacities such as strength, balance and the risk of falls in healthy elderly people, but the difference in static balance is not significant when comparing with the control groups. These results make us see the need to continue studying different modalities of interventions that can significantly improve balance in older adults. Further research should be carried out to analyze the effects of these interventions in the long terms and with larger sample sizes. The present study focuses on the static analysis of equilibrium, the results may differ if the dynamic equilibrium is analyzed.

## Figures and Tables

**Table 1:** Study characteristics.

Study	N (IG/CG)	Age (±SD)	Gender (F/M)	Modality	Variable
Wolfson, 1996 [31]	55 (28/27)	80	32/23	BT	OLS-CE
Wolfson, 1996 [31]	55 (28/27)	80	34/21	RT	OLS-CE
Earles, 2001 [32]	40 (18/22)	78 ± 5	26/14	RT	OLS
Kronhed, 2001 [34]	30 (15/15)	73 ± 2	16/14	BT	Romberg
Mian, 2007 [35]	38 (25/13)	73 ± 3.4	19/19	MCT	OLS
Kobayashi, 2012 [30]	24 (17/7)	67.5 ± 5.23	14/10	RT	OLS-CE
Bieryla, 2013 [36]	12 (6/6)	81.5 ± 5.5	10/2	BT	BBS
Sato, 2015 [33]	58 (29/28)	69.25 ± 5.41	43/14	BT	BBS
Marques, 2017 [39]	47 (24/23)	69	47/0	RT	OLS
Marques, 2017 [39]	48 (24/24)	69	48/0	AT	OLS
Adcock, 2020 [4]	31 (15/16)	73.9 ± 6.4	16/15	MCT	SPPB Balance

IG: intervention group; CG: control group; BT: balance training; RT: resistance training; MCT: multicomponent training; AT: aerobic training; M: male; F: female; SD: standard deviation; OLS: one-legged stance test; CE: closed eyes; BBS: Berg balance scale; SPPB: short physical performance battery.

**Table 2:** PEDro score.

Study	1	2	3	4	5	6	7	8	9	10	11	Total
Wolfson, 1996 [31]	1	1	0	1	0	0	1	1	0	1	1	6
Earles, 2001 [32]	1	1	0	1	0	0	0	1	0	1	1	5
Kronhed, 2001 [34]	1	1	1	1	0	0	0	0	1	1	1	6
Mian, 2007 [35]	1	1	0	1	0	0	0	0	0	1	1	4
Kobayashi, 2012 [30]	1	1	0	1	0	0	0	1	0	1	1	5
Bieryla, 2013 [36]	1	1	0	1	0	0	0	0	0	0	1	3
Sato, 2015 [33]	0	1	0	1	0	0	0	1	0	1	1	5
Marques, 2017 [39]	1	1	0	1	0	0	1	0	1	1	1	6
Adcock, 2020 [4]	1	1	1	1	0	0	0	0	0	1	1	5
Average												5 *

1: Inclusion/exclusion criteria; 2: random allocation of participants; 3: concealed allocation; 4: similarity between groups at baseline; 5: participant blinding; 6: therapist blinding; 7: assessor blinding; 8: fewer than 15% dropouts; 9: intention- to-treat analysis; 10: between-group statistical comparisons; 11: point measures and variability data. * Item 1 is not used to calculate the PEDro score.

## Data Availability

The data presented in this study are available on request from the corresponding author.

## Supplementary Materials

The following are available online at https://www.mdpi.com/article/10.3390/life13051193/s1, S1a: Keywords used for search strategy, S1b: complete literature search.

## References

[1] Lesinski M., Hortobágyi T., Muehlbauer T., Gollhofer A., Granacher U. (2015). Effects of Balance Training on Balance Performance in Healthy Older Adults: A Systematic Review and Meta-analysis. Sport Med..

[2] Labata-Lezaun N., González-Rueda V., Llurda-Almuzara L., López-de-Celis C., Rodríguez-Sanz J., Bosch J., Vicente-Rodríguez G., Gorczakowska D., Araluze-Arizti P., Pérez-Bellmunt A. (2023). Effectiveness of multicomponent training on physical performance in older adults: A systematic review and meta-analysis. Arch. Gerontol. Geriatr..

[3] Álvarez E., Alud A. (2018). La actividad física y sus beneficios físicos como estrategia de inclusión social del adulto mayor. Incl. Desarro..

[4] Adcock M., Fankhauser M., Post J., Lutz K., Zizlsperger L., Luft A.R., Guimarães V., Schättin A., De Bruin E.D. (2020). Effects of an In-home Multicomponent Exergame Training on Physical Functions, Cognition, and Brain Volume of Older Adults: A Randomized Controlled Trial. Front. Med..

[5] Gillespie L.D., Robertson M.C., Gillespie W.J., Sherrington C., Gates S., Clemson L., E Lamb S. (2012). Interventions for preventing falls in older people living in the community. Cochrane Database Syst. Rev..

[6] Mahoney J.R., Cotton K., Verghese J. (2019). Multisensory integration predicts balance and falls in older adults. J. Gerontol..

[7] López-de-Celis C., Zegarra-Chávez D., Cadellans-Arróniz A., Carrasco-Uribarren A., Izquierdo-Nebreda P., Canet-Vintró M., Rodríguez-Sanz J., Pérez-Bellmunt A. (2022). Study on Balance and Postural Control According to the Stabilometry in Indoor Skydivers: A Cross-Sectional Study. Int. J. Environ. Res. Public Health.

[8] Era P. (1988). Posture control in the elderly. Int. J. Technol. Aging.

[9] Moreland J.D., Richardson J.A., Goldsmith C.H., Clase C.M. (2004). Muscle weakness and falls in older adults: A systematic review and meta-analysis. J. Am. Geriatr. Soc..

[10] Bijlsma A.Y., Pasma J., Lambers D., Stijntjes M., Blauw G.J., Meskers C.G., Maier A. (2013). Muscle strength rather than muscle mass is associated with standing balance in elderly outpatients. J. Am. Med. Dir. Assoc..

[11] Garatachea N., Pareja-Galeano H., Sanchis-Gomar F., Santos-Lozano A., Fiuza-Luces C., Morán M., Emanuele E., Joyner M.J., Lucia A. (2015). Exercise attenuates the major hallmarks of aging. Rejuven. Res..

[12] Aragao F.A., Karamanidis K., Vaz M.A., Arampatzis A. (2011). Mini-trampoline exercise related to mechanisms of dynamic stability improves the ability to regain balance in elderly. J. Electromyogr. Kinesiol..

[13] Littbrand H., Lundin-Olsson L., Gustafson Y., Rosendahl E. (2009). The Effect of a High-Intensity Functional Exercise Program on Activities of Daily Living: A Randomized Controlled Trial in Residential Care Facilities. J. Am. Geriatr. Soc..

[14] Maitre J., Jully J.L., Gasnier Y., Paillard T. (2013). Chronic physical activity preserves efficiency of proprioception in postural control in older women. J. Rehabil. Res. Dev..

[15] Patti A., Bianco A., Paoli A., Messina G., Montalto M.A., Bellafiore M., Battaglia G., Iovane A., Palma A. (2015). Effects of pilates exercise programs in people with chronic low back pain: A systematic review. Medicine.

[16] Casas-Herrero Á., de Asteasu M.L.S., Antón-Rodrigo I., Sánchez-Sánchez J.L., Montero-Odasso M., Marín-Epelde I., Ramón-Espinoza F., Zambom-Ferraresi F., Petidier-Torregrosa R., Elexpuru-Estomba J. (2022). Effects of Vivifrail multicomponent intervention on functional capacity: A multicentre, randomized controlled trial. J. Cachexia Sarcopenia Muscle.

[17] Izquierdo M., Merchant R.A., Morley J.E., Anker S.D., Aprahamian I., Arai H., Aubertin-Leheudre M., Bernabei R., Cadore E.L., Cesari M. (2021). International Exercise Recommendations in Older Adults (ICFSR): Expert Consensus Guidelines. J. Nutr. Health Aging.

[18] Sherrington C., Michaleff Z.A., Fairhall N., Paul S.S., Tiedemann A., Whitney J., Cumming R.G., Herbert R.D., Close J.C.T., Lord S.R. (2017). Exercise to prevent falls in older adults: An updated systematic review and meta-analysis. Br. J. Sport. Med..

[19] Brox E., Luque L.F., Evertsen G.J., Hernández J.E.G. Exergames for elderly: Social exergames to persuade seniors to increase physical activity. Proceedings of the 2011 5th International Conference on Pervasive Computing Technologies for Healthcare (PervasiveHealth) and Workshops.

[20] Aramaki A.L., Sampaio R.F., Reis A.C., Cavalcanti A., Dutra F.C. (2019). Virtual reality in the rehabilitation of patients with stroke: An integrative review. Arq. Neuropsiquiatr..

[21] Laufer Y., Dar G., Kodesh E. (2014). Does a Wii-based exercise program enhance balance control of independently functioning older adults? A systematic review. Clin. Interv. Aging.

[22] Page M.J., McKenzie J.E., Bossuyt P.M., Boutron I., Hoffmann T.C., Mulrow C.D., Shamseer L., Tetzlaff J.M., Akl E.A., Brennan S.E. (2021). The PRISMA 2020 statement: An updated guideline for reporting systematic reviews. BMJ.

[23] Methley A.M., Campbell S., Chew-Graham C., McNally R., Cheraghi-Sohi S. (2014). PICO, PICOS and SPIDER: A comparison study of specificity and sensitivity in three search tools for qualitative systematic reviews. BMC. Health Serv. Res..

[24] Cumpston M., Li T., Page M., Chandler J., Welch V., Higgins J.P., Thomas J. (2019). Cochrane Handbook for Systematic Reviews of Interventions.

[25] Ouzzani M., Hammady H., Fedorowicz Z., Elmagarmid A. (2016). Rayyan-a web and mobile app for systematic reviews. Syst. Rev..

[26] Cashin A.G., McAuley J.H. (2020). Clinimetrics: Physiotherapy Evidence Database (PEDro) Scale. J. Physiother..

[27] Sterne J.A.C., Savović J., Page M.J., Elbers R.G., Blencowe N.S., Boutron I., Shamseer L., Tetzlaff J.M., Akl E.A., Brennan S.E. (2019). RoB 2: A revised tool for assessing risk of bias in randomized trials. BMJ.

[28] DerSimonian R., Laird N. (1986). Meta-analysis in clinical trials. Control Clin. Trials.

[29] Higgins J.P.T., Thompson S., Deeks J., Altman D. (2003). Measuring inconsistency in meta-analyses. BMJ.

[30] Kobayashi H., Koyama Y., Enoka R.M., Suzuki S. (2014). A unique form of light-load training improves steadiness and performance on some functional tasks in older adults. Scand. J. Med. Sci. Sport.

[31] Wolfson L., Whipple R., Derby C., Judge J., King M., Amerman P., Schmidt J., Smyers D. (1996). Gains and Tai Chi Maintenance. Am. Geriatr. Soc..

[32] Earles D.R., Judge J.O., Gunnarsson O.T. (2001). Velocity training induces power-specific adaptations in highly functioning older adults. Arch. Phys. Med. Rehabil..

[33] Sato K., Kuroki K., Saiki S., Nagatomi R. (2015). Improving Walking, Muscle Strength, and Balance in the Elderly with an Exergame Using Kinect: A Randomized Controlled Trial. Games Health J..

[34] Kronhed A.-C.G., Möller C., Olsson B., Möller M. (2001). The Effect of Short-Term Balance Training on Community-Dwelling Older Adults. J. Aging Phys. Act..

[35] Mian O., Thom J., Ardigò L., Morse C., Narici M., Minetti A. (2007). Effect of a 12-month physical conditioning programme on the metabolic cost of walking in healthy older adults. Eur. J. Appl. Physiol..

[36] Bieryla K.A. (2013). Dold NM. Feasibility of Wii Fit training to improve clinical measures of balance in older adults. Clin. Interv. Aging.

[37] Cadore E.L., Casas-Herrero A., Zambom-Ferraresi F., Idoate F., Millor N., Gómez M., Rodríguez-Mañas L., Izquierdo M. (2014). Multicomponent exercises including muscle power training enhance muscle mass, power output, and functional outcomes in institutionalized frail nonagenarians. Age.

[38] Forte R., Boreham C.A.G., Leite J.C., De Vito G., Brennan L., Gibney E.R., Pesce C. (2013). Enhancing cognitive functioning in the elderly: Multicomponent vs resistance training. Clin. Interv. Aging.

[39] Marques E.A., Figueiredo P., Harris T.B., Wanderley F.A., Carvalho J. (2017). Are resistance and aerobic exercise training equally effective at improving knee muscle strength and balance in older women?. Arch. Gerontol. Geriatr..

[40] Knerl C., Schuler P., Taylor L., Cosio-Lima L., Caillouet K. (2009). The effects of six weeks of balance and strength training on measures of dynamic balance of older adults. Calif. J. Health Promot..

[41] Motalebi S.A., Cheong L.S., Iranagh J.A., Mohammadi F. (2018). Effect of low-cost resistance training on lower-limb strength and balance in institutionalized seniors. Exp. Aging Res..

[42] Sobrinho A.C.d.S., Almeida M.L.d., Rodrigues G.d.S., Finzeto L.C., Silva V.R.R., Bernatti R.F., Bueno Junior C.R. (2021). Effect of Flexibility Training Associated with Multicomponent Training on Posture and Quality of Movement in Physically Inactive Older Women: A Randomized Study. Int. J. Environ. Res. Public Health.

[43] Schlicht J., Camaione D.N., Owen S.V. (2001). Effect of intense strength training on standing balance, walking speed, and sit-to-stand performance in older adults. J. Gerontol. A Biol. Sci. Med. Sci..

[44] Fragala M.S., Cadore E.L., Dorgo S., Izquierdo M., Kraemer W.J., Peterson M.D., Ryan E.D. (2019). Resistance Training for Older Adults: Position Statement From the National Strength and Conditioning Association. J. Strength Cond. Res..

[45] Sadjapong U., Yodkeeree S., Sungkarat S., Siviroj P. (2020). Multicomponent Exercise Program Reduces Frailty and Inflammatory Biomarkers and Improves Physical Performance in Community-Dwelling Older Adults: A Randomized Controlled Trial. Int. J. Env. Res. Public Health.

[46] Pahor M., Guralnik J.M., Ambrosius W.T., Blair S., Bonds D.E., Church T.S., Espeland M.A., Fielding R.A., Gill T.M., Groessl E.J. (2014). Effect of structured physical activity on prevention of major mobility disability in older adults: The LIFE study randomized clinical trial. JAMA.

[47] López J.C., Arango E.F. (2015). Efectos del entrenamiento en superficies inestables sobre el equilibrio y funcionalidad en adultos mayores. Rev. Fac. Nac. Salud. Pública..

[48] Javadpour S., Sinaei E., Salehi R., Zahednejad S., Motealleh A. (2022). Comparing the Effects of Single-Task versus Dual-Task Balance Training on Gait Smoothness and Functional Balance in Community-Dwelling Older Adults: A Randomized Controlled Trial. J. Aging Phys. Act..

[49] Sinaei E., Kamali F., Nematollahi A., Etminan Z. (2016). Comparing the effects of balance training with and without cognitive tasks on the quality of life and balance performance in community-dwelling older adults: A single-blind randomized clinical trial. J. Rehabil. Sci. Res..

[50] Ferguson B. (2014). ACSM’s Guidelines for Exercise Testing and Prescription 9th Ed. 2014. J. Can. Chiropr. Assoc..

[51] Spagnuolo D.L., Jurgensen S.P., Iwama A.M., Dourado V.Z. (2010). Walking for the assessment of balance in healthy subjects older than 40 years. Gerontology.

[52] Shigematsu R., Chang M., Yabushita N., Sakai T., Nakagaichi M., Nho H. (2002). Dance-based aerobic exercise may improve indices of falling risk in older women. Age Ageing.

[53] Vidarte-Claros J.A., Quintero-Cruz M.V., Herazo-Beltrán Y. (2012). Efectos del ejercicio físico en la condición física funcional y la estabilidad en adultos mayores. Hacia Promoc. Salud.

[54] Hayashi D., Gonçalves C.G., Parreira R.B., Fernandes K.B., Teixeira D.C., Silva R.A., Probst V.S. (2012). Postural balance and physical activity in daily life (PADL) in physically independent older adults with different levels of aerobic exercise capacity. Arch. Gerontol. Geriatr..

