# Supplementary material for: Effectiveness of Different Training Modalities on Static Balance in Older Adults: A Systematic Review and Meta-Analysis
Source: Life (Basel). 2023 May 16;13(5):1193. doi: 10.3390/life13051193 (PMC10222379; doi:10.3390/life13051193)
Supplement: Supplementary file 1 [file life-13-01193-s001.zip › life-2303164-supplementary.pdf]

Supplementary Material S1a. Keywords used for the search strategy.

Population	Intervention	Outcomes
Aged	Resistance training	Balance
Old people	Strength training	Proprioception
Elderly	Weightlifting	
Aging	Endurance training	
Older people	Aerobic training	
Older adults	Walking	
Old adults	Balance training	
Senior	Multicomponent training	
Geriatric	Multicomponent training	
	Multicomponent exercise	

Supplementary Material S1b: Complete literature search.

PubMed Search Formula

(aged OR "old people" [Title] OR "older people" [Title] OR "older adults" [Title] OR "old adults" [Title] OR elderly OR senior [Title] OR geriatric [Title]) AND ("randomized controlled trial"[Publication Type] OR "controlled clinical trial"[Publication Type] OR "randomized"[Title/Abstract] OR "placebo"[Title/Abstract] OR "clinical trials as topic"[MeSH Terms] OR "randomly"[Title/Abstract] OR "trial"[Title]) AND (balance OR proprioception) AND ("aerobic training" OR "endurance training" OR walking OR "balance training" OR "resistance training" OR "strength training" OR weightlifting OR "multicomponent training" OR "multi component training" OR "multi-component training" OR "multicomponent exercise")

Results: 338

Date: 14/01/2023

Cochrane Library Search Formula

(aged OR "old people" OR "older people" OR "older adults" OR "old adults" OR elderly OR senior OR geriatric) in Title Abstract Keyword AND ("aerobic training" OR "endurance training" OR walking OR "balance training" OR "resistance training" OR "strength training" OR weightlifting OR "multicomponent training" OR "multi component training" OR "multi-component training" OR "multicomponent exercise") in Title Abstract Keyword AND (balance OR proprioception)

Results: 258

Date: 14/01/2023

Web of Science Search Formula

#1

TI=(aged OR "old people" OR "older people" OR "older adults" OR "old adults" OR elderly OR senior OR geriatric)

#2

TS=("aerobic training" OR "endurance training" OR walking OR "balance training" OR "resistance training" OR "strength training" OR weightlifting OR "multicomponent trainig" OR "multi component training" OR "multi-component training" OR "multicomponent exercise")

#3

TS=(balance OR proprioception)

#4

TS=research design OR TS=comparative stud* OR TS=evaluation stud* OR TS=controlled trial* OR TS=follow-up stud* OR TS=prospective stud* OR TS=random* OR TS=placebo* OR TS=(single blind*) OR TS=(double blind*)

#5 (TS=#1 AND #2 AND #3 AND #4)

Results: 363

Date: 14/01/2023

SportDiscus Search Formula

#1 Título

(aged OR "old people" OR "older people" OR "older adults" OR "old adults" OR elderly OR senior OR geriatric)

#2

("aerobic training" OR "endurance training" OR walking OR "balance training" OR "resistance training" OR "strength training" OR weightlifting OR "multicomponent training" OR "multi component training" OR "multi-component training" OR "multicomponent exercise")

#3 (balance OR proprioception)

Results: 144

Date: 14/01/2023
